# Case report: Ultrasound-guided multi-site electroacupuncture stimulation for a patient with spinal cord injury

**DOI:** 10.3389/fneur.2022.903207

**Published:** 2022-08-24

**Authors:** Xi-Zi Song, Xiao-Lei Chu, Tao Liu, Yu-Tong Cao, Rui-Xin Li, Ming-Wei Gao, Qing-Wen Li, Xiao-Song Gu, Dong Ming

**Affiliations:** ^1^Academy of Medical Engineering and Translational Medicine, Tianjin University, Tianjin, China; ^2^Department of Rehabilitation, Tianjin University Tianjin Hospital, Tianjin, China; ^3^College of Exercise & Health Sciences, Tianjin University of Sport, Tianjin, China; ^4^College of Precision Instruments and Optoelectronics Engineering, Tianjin University, Tianjin, China

**Keywords:** spinal cord injury, nerve, electroacupuncture stimulation, ultrasound-guided, case report

## Abstract

**Introduction:**

Spinal cord injury causes permanent neurological deficits, which have devastating physical, social, and vocational consequences for patients and their families. Traditional Chinese medicine uses acupuncture to treat neuropathic pain and improve nerve conduction velocity. This treatment can also reduce peripheral nerve injury joint contracture and muscle atrophy in affected patients. And it's got a remarkable restoration when electrical stimulation therapy on impaired peripheral nerves in animal models and clinical trials.

**Case description:**

A 48-year-old woman was hit by a heavy object that injured her lower back. The patient had a T12-L1 vertebral flexion and stretch fracture with traumatic spinal stenosis. The patient was transferred to the rehabilitation department after posterior T12-L2-segment pedicle screw system distraction and reduction, internal fixation, decompression, and bone graft fusion. Ultrasound-guided electroacupuncture was used to stimulate the sacral nerve, the spinal nerve, and the head of the patient, accompanied by spinal joint loosening training, respiratory training, lumbar comprehensive sports training, paraplegic limbs comprehensive training, and other manipulative treatment.

**Outcomes:**

After the intervention, the patient showed significant improvements in sensory and motor scores, resulting in functional recovery according to ASIA and FIM. The patient gradually showed reasonable functional remission.

**Discussion:**

The sacral nerve, the spinal cord, and the head were electrically stimulated by ultrasound-guided electroacupuncture in terms of intervention, and various functions of the patient were alleviated to a certain extent. The efficacy of ultrasound-guided electroacupuncture stimulation in treating neurologic symptoms should be validated in future clinical trials.

## Introduction

Spinal cord injury (SCI) is an acute injury that occurs in response to an external physical impact, such as a motor vehicle injury, sports-related injury, or violence (1). The spinal cord has poor potential for internal recovery and is, therefore, prone to permanent neurological damage. Furthermore, patients with spinal cord injury can have significant physical issues, owing to bladder dysfunction and urinary incontinence (UI). The major secondary lesions of death in patients with SCI are renal failure and urinary sepsis. Although survival rates for patients with SCI have improved over time, the mortality rate in these patients is higher than that in an age-matched control group (1). Mortality increases with the severity and severity of the injury (i.e., cervical spine trauma has a higher mortality rate than lumbar trauma), age of the patient, and presence of multi-system injuries and high-energy injury mechanisms. In addition, bladder dysfunction and urinary incontinence impose a heavy physiological burden on patients with SCI. Renal failure and uremia caused by SCI are the main causes of death in these patients even after recovery from the initial injury.

When conservative treatment fails, sacral neuromodulation (SNM) may well be used for the treatment of pelvic urinary indications and neurogenic bladder. According to data from animal and human pilot studies, the procedure is minimally invasive and carries low risk. Bladder compliance and bladder volume will be maintained through this treatment. In the same way, urinary tract infections will be reduced in acute situations following SCI (2, 3). Epidural electrical stimulation (EES) enables rodent, feline, and non-human primate models of leg paralysis to stand, walk in various directions, and run. This is because treatment can make the executive centers coordinate a wide range of motor behaviors immediately when it is applied to the lumbar spinal cord (4). In the clinic, EES is less used than other techniques. The main reason is that, in EES, an implantable electrode enclosed within a catheter is implanted into patients. Therefore, patients need a long recovery time, and the incidence of postoperative complications and treatment costs are also increased. The rate of postoperative complications is as high as 35–40% when patients receive the treatment according to related research (5). Transcranial current stimulation (TCS) is a non-invasive brain stimulation technique that involves applying a persistent, low-intensity electrical current over the scalp. Anodal TCS is an excellent treatment that can help the brain reach an optimal state of excitability, speed up motor learning, and accelerate training effects for spinal cord patients (6, 7), because anodal stimulation increases cortical excitability. However, its low focalization and high cost have limited its use as a standard and continuous treatment (8). Furthermore, the treatment-related expenses are expensive. Approximately, one-third patients will develop complications, and part of the complications will require huge treatment costs. So, we need to find a cheap and easy way to simulate SNM, EES, and TCS in patients with spinal cord injury (9).

Acupuncture, which originates from traditional Chinese medicine, can improve nerve conduction velocity and alleviate neuropathic pain (10, 11). It can improve the condition of patients who have muscle wasting and joint contracture due to peripheral nerve injuries (12). The results of electrical stimulation when used to treat injured nerves in animal and human models have been satisfactory (13, 14).

Electroacupuncture (EA) is a simple and effective treatment approach for SCI. Based on relevant research, after rat SCI, the effects of acupuncture on dorsal neuron function and neuroprotection are important. EA stimulation can promote neuronal functional recovery greatly when it acts on SDU26 and DU16 (Shuigou and Fengfu). Acupuncture's antioxidation, anti-inflammatory, and antiapoptotic effects are likely responsible for these improvements (15). But the selection of EA electrode targets usually depends on traditional Chinese medicine, and does not consider the properties of electrical transmission. Furthermore, in previous studies, 1.3% nerve injuries were caused by acupuncture, so improving the precision is crucial (16, 17).

This study examines the efficacy and outcome of ultrasound-guided EA stimulation in a patient with SCI who did not respond to general rehabilitation exercises, such as spinal joint loosening training, breathing training, lumbar-integrated movement training, and paraplegic-integrated limb training.

## Case description

### History

**Figure 1:**
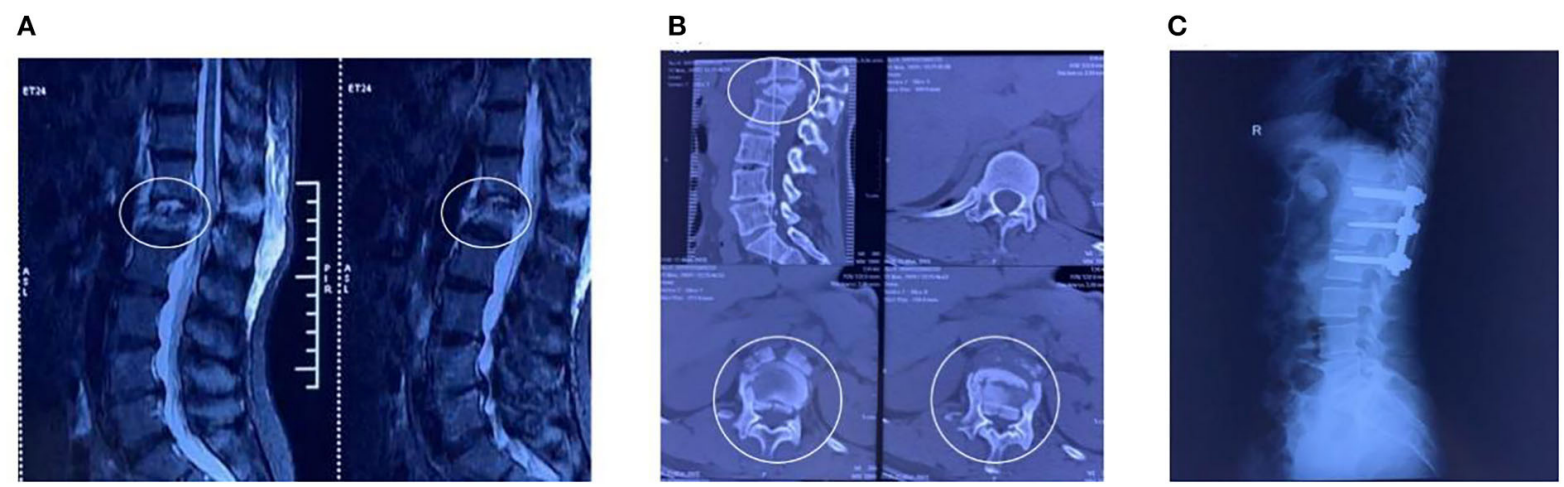
MR images and Radiographic before treatment. **(A,B)** Lumbar magnetic resonance imaging and computed tomography showed explosive L1 fracture with severe SCI. **(C)** Postoperative radiograph.

The patient was a 48-year-old woman who sustained a lower-back injury after being hit by a heavy object. She immediately experienced pain in her lower back and loss of sensation in her lower limbs and was unable to move. Upon admission, she was diagnosed with T12-L1 vertebral flexion and stretch fracture accompanied by traumatic spinal stenosis (Figures 1A,B). The final diagnosis was SCI with paraplegia (The American Spinal Injury Association impairment scale: B). Combined with physical examination and imaging, no obvious contraindications were found in various laboratory tests. The posterior thoracic T12-L2 pedicle screw system was used for distraction reduction, internal fixation, lamina decompression, and bone graft fusion (Figure 1C).

The patient was transferred to the rehabilitation department 2 weeks after the operation for manipulation therapy, including spinal joint loosening training, breathing training, lumbar-integrated exercise training, and paraplegic limb comprehensive training. However, the patient's symptoms did not significantly improve. To improve the quality of life and self-care ability, she opted for acupuncture.

### Diagnosis

The patient underwent a detailed clinical examination after her first visit. No obvious abnormalities were observed in each joint of both lower extremities. To evaluate the muscle strength of each key muscle in both lower limbs, muscle fiber fibrillation can be seen in the iliopsoas muscle and the quadriceps muscle eye, but no movement can be formed. The tibialis anterior, extensor, and gastrocnemius muscles were completely paralyzed, and muscle contractions were invisible or undetectable. Blood circulation in both lower extremities was somewhat poor. Taking into account the previous baseline, we decided to apply a different intervention. Informed consent was obtained from the patient for publication of this case report.

#### Intervention

The intervention proposed included stimulation of the sacral nerve with ultrasound-guided EA, combined with a water-drinking program and intermittent catheterization to improve urinary retention in the bladder; furthermore, ultrasound-guided EA stimulation of the spinal cord and head could improve muscular dystonia in the lower extremity.

The application of ultrasound-guided EA stimulation was based on anatomical structure. In this case, an electrical current was applied to the acupuncture needles placed close to the nerve. Using ultrasound imaging, peripheral nerves could be visualized based on relevant research. Invasive therapies like neural blocks can be made more precise and accurate using this technology (18).

### Ultrasound-guided EA stimulation of the sacral nerve

**Figure 2:**
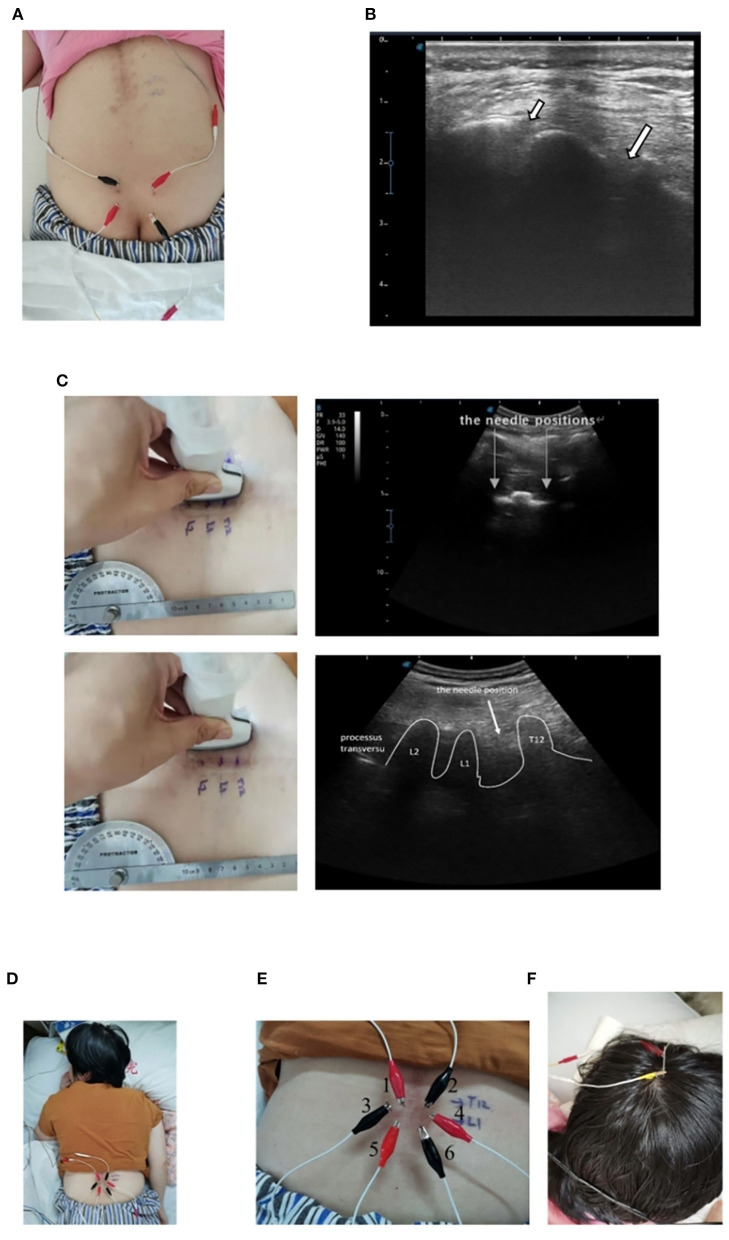
Schematic of EA stimulation. **(A)** Sacral nerve EA stimulation on the patient. **(B)** Ultrasonographic images of the S4 (a short arrow) and S3 (a long arrow) foramen. **(C)** Ultrasonographic images of the T12 to the L2 vertebrae. **(D)** Spinal nerve EA stimulation on the patient. **(E)** EA distribution of spinal nerve stimulation. **(F)** EA stimulation of the head on the patient.

After spinal joint looseness training, respiratory training, lumbar-integrated exercise training, and paraplegic limb training, EA stimulation of the sacral nerve was performed. In 2005, an article described a case report of sacral nerve stimulation, which located S3 and S4 nerves for electrical stimulation to treat neurogenic bladder urinary retention (19). According to that article, the four therapeutic EA electrodes (HUA CHENG.30 mm × 75 mm) were located in the S3 and S4 foramen connected with an electro stimulator (sd Z-III electroacupuncture instrument, Hwato brand, China) at 20 Hz with 220 ms wave width, which is similar with the sacral nerve stimulator (Figures 2A,B). The amount of stimulation was increased until the patient developed flexion reflex of the great toe or rectal traction (20, 21). And the range of intensity was 100-120 mV, five times a week; the treatment was terminated until the patient's residual urine volume was maintained below 100 ml. Each stimulation lasted about 1 h, supplemented by a water-drinking program and intermittent urethral catheterization. Daily drinking time and quantity were fixed.

### Ultrasound-guided EA stimulation of the spinal cord and the head

We used a Doppler ultrasonic-diagnostic apparatus (SonoScape^®^, China) at 12 MHz. The spinal nerve was imaged in transverse cross-sectional (a short axis) and longitudinal (a long axis) views between T12 and L1 to locate the electrical stimulation (Figure 2C). We used a kind of acupuncture needle for carrying an electric current (HUA CHENG.30 mm × 40 mm). The first and second needles were located between T12 and L1. The third and fourth needles were located at the left and right nerve roots of L1, and the fifth and sixth needles were located between L1 and L2. The six needles were arranged in a hexagonal pattern to create an electric field stimulus at the affected area (Figure 2D). The depth of EA is about 3.5 cm, not more than 1/2 of the ligamentum flavum. When inserting the EA electrode, it is necessary to prevent it from passing through the ligamentum flavum and causing secondary injury to the spinal cord. Six needles were left in place at six points connected to an electro stimulator (SdZ-III electronic acupuncture therapy instrument, Hwato brand, China) to apply a continuous waveform, at 50 Hz and with 220-ms wave width for 1 h (22) (Figure 2E). The intervention was repeated five times/week until discharge.

The patient received another intervention stimulation of the head during the session. The EA electrode was inserted 1.5 cm in front of the central sulcus and extended along with the skull to the front of the forehead (Figure 2F). The program was performed five times/week.

#### Outcomes

The results of nearly a 1-year period are presented in this report. A significant improvement in the patient's condition was noted after treatment.

**Figure 3:**
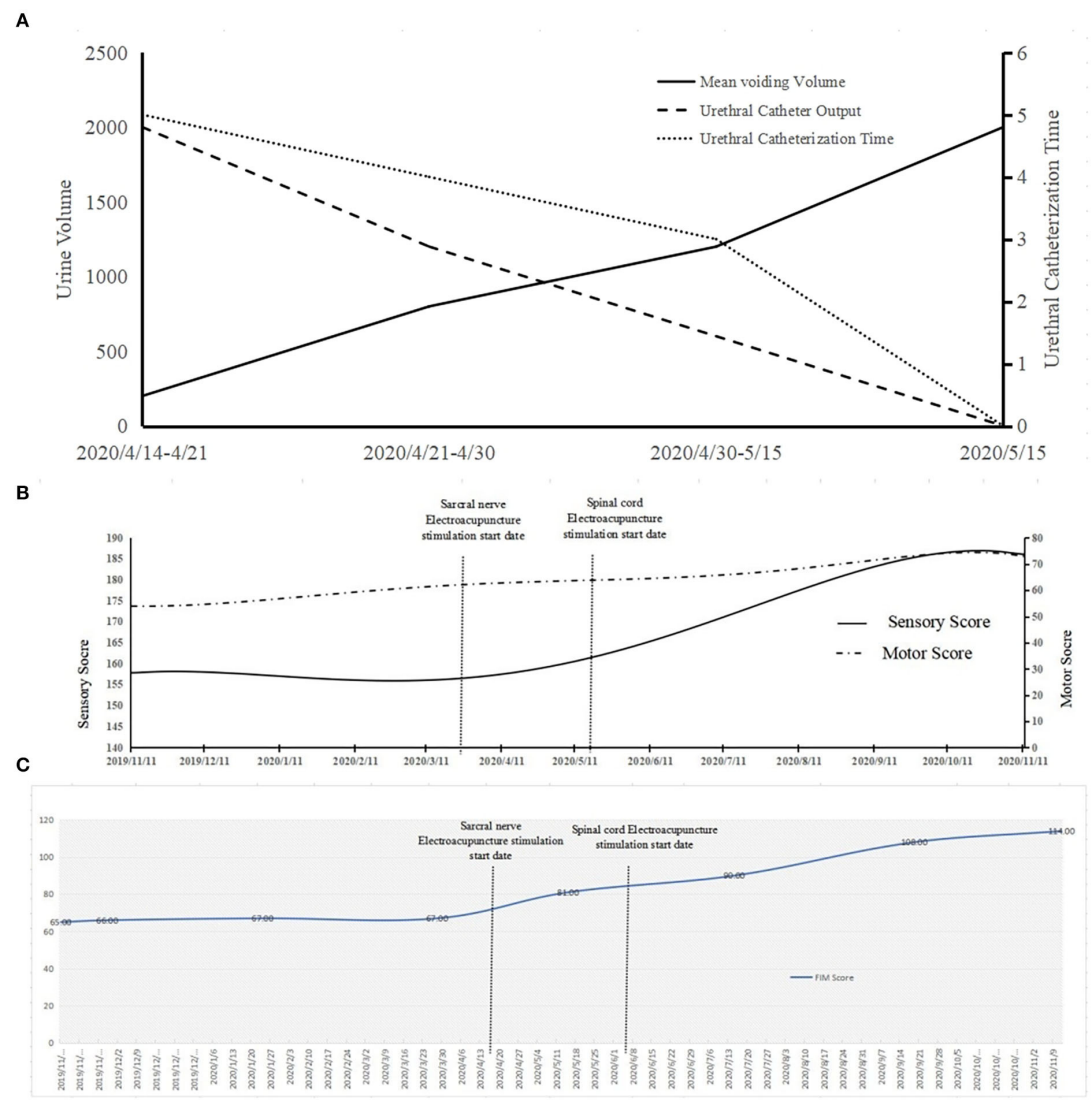
The changes of the patient's function during the study period. **(A)** The results show the voiding volume and the volume of the urethral catheter. The right coordinate axis shows the voiding volume and urethral catheter output. The left axis displays the times of urethral catheterization times. **(B)** The ASIA score curve of the patient. **(C)** The FIM score curve of the patient.

For urinary retention in the bladder, we simultaneously applied clean intermittent catheterization and sacral nerve EA stimulation. We found that voiding volume by the patient herself has been increasing; meanwhile, the volume of the urethral catheter decreases. Hence, we reduced the urethral catheterization times from five to zero. After the urethral catheter was removed, the residual urine volume was detected, which was only 80 ml, proving that urinary retention in the bladder had improved (Figure 3A).

**Figure 4:**
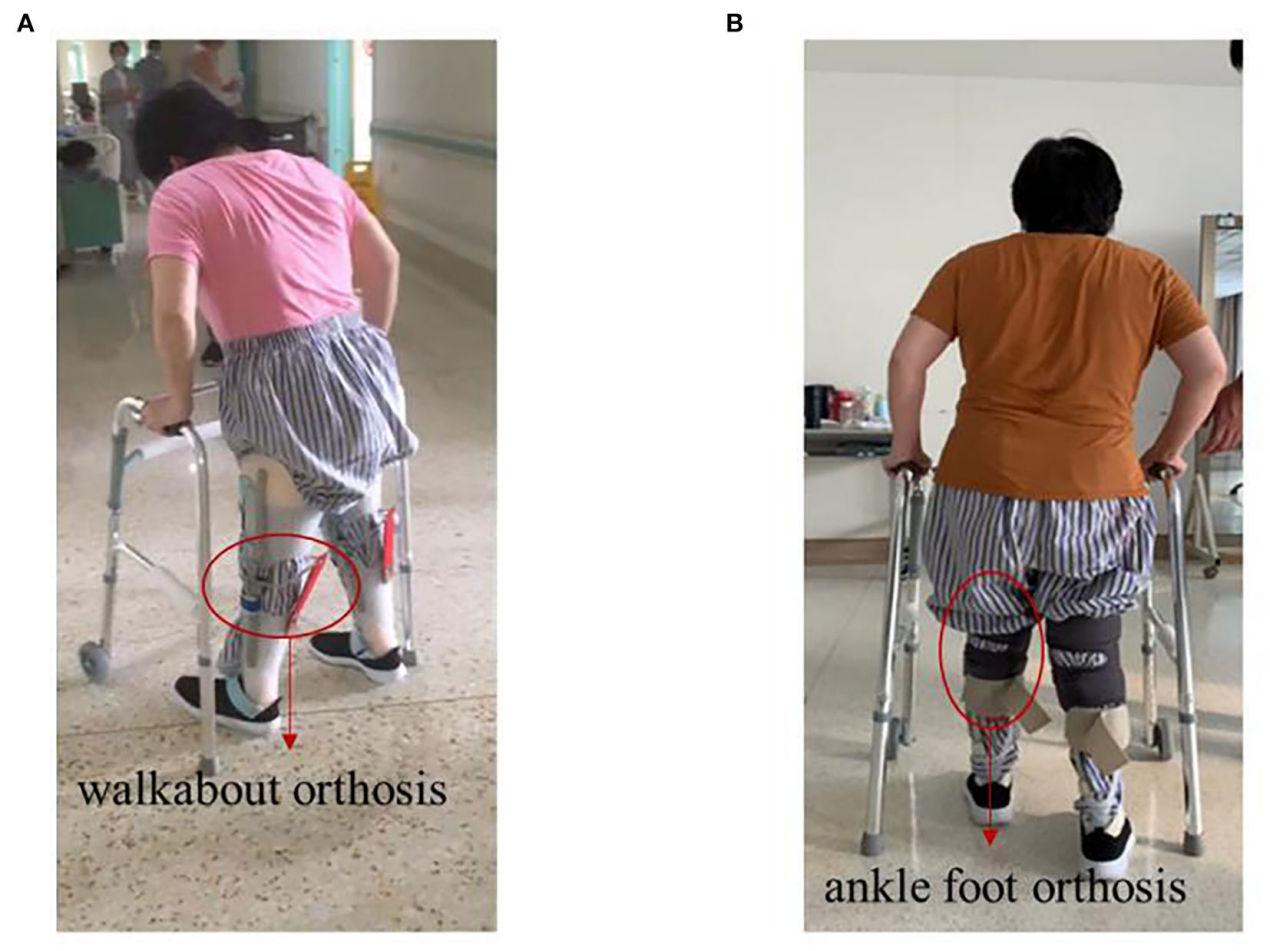
Photos of the patient walking before and after treatment. **(A)** Before treatment, walk with the help of walkabout orthosis. **(B)** After treatment, walk with the help of ankle-foot orthosis.

In terms of lower-limb muscle tone, the patient could only walk with a Walkabout orthosis in May 2020 (Figure 4A); she was able to walk with an ankle-foot orthosis in September 2020 (Figure 4B). Next, we compared the neurological level according to the American Spinal Injury Association (ASIA) scale (Supplementary Table 1). We did not find any difference between the ASIA neurological level and the sensory level, but we did find that both the impairment scale and the motor level were better than before. We further compared the scores from the ASIA scale from November 28, 2019 to November 11, 2020. The ASIA scale showed that there was no significant increase in the sensory score and the motor score from the beginning of rehabilitation to the beginning of sacral electrical stimulation. There were no significant changes in sensory scores (156 to 160) when sacral electrical stimulation was initiated. However, the motor scores increased from 58 to 64 after sacral electrical stimulation. When a combination of the spinal cord and head stimulation was added, the motor score (64 to 73) and the sensory score (160 to 186) both showed significant change (Figure 3B).

The results of comparison of the motor score and the sensory score suggested function recovery speed. We found that the slope value of the motor score showed little difference before and after spinal cord stimulation (Supplementary Table 2). However, the sensory score increased significantly after spinal cord stimulation. In the FIM score, the value did not change significantly before sacral EA stimulation (67 to 81) but increased significantly (81 to 114) after sacral EA stimulation (Figure 3C). The slope value increased slowly at first, but its increase rate was rapid after the sacral stimulation. The rate did not slow down with increasing time after spinal cord stimulation (Supplementary Table 3). The imaging data of patient after rehabilitation in Supplementary Figure 1.

## Discussion

The physical therapy methods and clinical reasoning of a patient with traumatic SCI are described in this case report. According to the clinical examination, ultrasound-guided EA stimulation was used to target the nerve in this patient. This is a new type of intervention approach targeting the nerve. The stimulation points we selected and the traditional Chinese medicine acupoints have some similarities. For example, the position for the S3 and S4 foramen is similar to Baliao (23, 24). However, compared with acupoints, the points we selected based on the principles of EA stimulation of the sacral nerve, and the stimulation position used in our patient was deeper and more precise.

Sacral nerve EA for the treatment of urinary retention is an improvement based on SNM in our case. The mechanism of sacral nerve EA in the treatment of urinary retention is unclear, but it is similar to the mechanism of SNM in the treatment of SCI, that is, the bladder responds to nerve stimulation with an initial rapid contraction, followed by a slow, sustained relaxation. Shi et al. (25) revealed that SNM could decrease uninhibited detrusor contractions and peak bladder pressures during bladder filling in an experimental animal model of complete SCI. An animal model of complete spinal cord transection caused a decrease in β-adrenergic relaxation responses, and the results were shown to be mutated by SNM *in vitro* (26). The clinical results from humans demonstrated the effects of SNM. All patients who received SNM experienced incontinence, compared to 100% of the control group. The patients with SNM had fewer UTIs (.5/year vs. 3.8/year) and several readmissions. However, the limitation of SNM is obvious. Pain at the implant site and lead migration were the most common adverse events reported. Another health technology investigated the costing factors of patients to SNM. This investigation showed that the cost-effectiveness between SNS and incontinence supplies is essentially identical. This can be attributed to the adjustments required to make the device most effective and the cost of the procedure. SNS also had a positive impact on the quality of life (27).

In traditional Chinese medicine, the sacral nerve is stimulated with acupuncture needles through the S3 and S4 foramen for the treatment of urinary tract diseases. This way, we used the acupuncture needle to module SNS based on its basic electrical stimulation parameters. The ultrasound-guided EA also increased the voiding volume and reduced the volume of the urethral catheter (Figure 3A). Despite it is not as effective as Bladder Stumilator it is. Compared with sacral nerve electrical stimulation, EA is minimally invasive and avoids the risk of secondary surgery due to battery depletion and electrode movement. Overall, its effects are very promising, and the cost of operation is low. We aim to explore the optimum parameters of the electrical needle in future studies. This treatment approach will likely be relevant and helpful for people with SCI with heavy economic burden, especially in undeveloped countries.

Epidural electrical stimulation is an emerging method for treating SCIs. Human studies have observed that EES triggers alternating rhythmic muscle activity patterns (28, 29), with a reduction in spasticity among patients with incomplete SCI and an increase in the amplitude of voluntary movements in paralyzed limbs (30). Spinal electrical stimulation using electrodes implanted in the epidural space is used to treat SCI (31). However, spinal epidural stimulation may lead to infection of the incision after surgery, spinal epidural hematoma, rejection of the implanted electrode, and a high cost of treatment. Owing to its minimally invasive nature, ultrasound-guided EA stimulation intervention used in our patient reduces the risk. Studies have shown that TCS has a significant effect on motor function in patients with SCI when compared to sham stimulation (32). Furthermore, some studies on transcranial electrostimulation in rats have shown that peripheral craniospinal sensory nerves play a significant role in mediating pulsed electrical stimulation's antinociceptive effects (33). In this study, the antinociceptive effect of stimulation was blocked by local anesthetics applied to the stimulation electrodes injected subcutaneously. Based on these results, it appears that the effects of low-intensity intracranial AC stimulation may be enhanced in brainstem centers by stimulating peripheral cranial nerves (CN1 through CN7), as well as craniospinal nerves (C1–C3) (34). We used the needle inserted into the scalp (precentral gyrus of body surface projection) to stimulate the CNS *via* the peripheral nervous system. The activation from the brain and the brainstem will then be transmitted to the lower motor neurons in the spinal cord, forming a combined effect with electrical stimulation of the spinal cord.

Although data from randomized clinical trials are necessary to further explore the clinical effects of ultrasound-guided EA, in this case report, we present preliminary evidence regarding the potential effectiveness of ultrasound-guided EA stimulation. It is interesting to note that the patient had been injured for 6 months at the time of initiating EA treatment, which is well beyond the prime time of treatment. However, after the first course of treatment, the patient reported regaining motor function, meaning that ultrasound-guided EA stimulation could produce a beneficial effect on both the motor and sensory systems.

This report has some limitations. First, although a few case series have shown promising effects of ultrasound-guided EA stimulation for subjects with neurogenic bladder retention and lower limb dystonia in patients with SCI, this intervention has not been sufficiently studied in literature. It should be noted that some discrepancies in the methodology, especially the material and insertion depth of acupuncture needles, and the duration of the electrical stimulation can be observed between previous studies and the current one—for example, Wilson et al. implanted permanent electrodes near the nerve (35). Second, the application parameters of electrical stimulation refer to the corresponding parameters of percutaneous electrical stimulation, but the best parameters need to be determined by an active electrical stimulation simulation experiment. Third, neither of the procedures have any psychometric properties (positive or negative likelihood ratios, specificity, or sensitivity data). To further identify the effectiveness of ultrasound-guided EA stimulation in lower limb dystonia, randomized controlled trials need to be conducted. Fourth, because this is a single case report, we eliminated the effects of chance. Randomized controlled trials with large sample size are needed to further determine the efficacy of ultrasound-guided EA in the treatment of dystonia of the lower extremity.

## Conclusions

This case report describes the successful rehabilitation of a patient with SCI. Physical therapy intervention included the use of ultrasound-guided EA on S3 and S4 nerves, supplemented by a water-drinking plan and intermittent urethral catheterization, and the use of spinal electroacupuncture on T12-L1, combined with anterior central EA therapy of the head. After 10 months of treatment, there were significant improvements in sensory conduction, nerve function, and muscle strength, and clinically significant changes in the patient's functional status.

## Data availability statement

The original contributions presented in the study are included in the article/Supplementary material, further inquiries can be directed to the corresponding authors.

## Ethics statement

The studies involving human participants were reviewed and approved by Medical Ethics Committee of Tianjin Hospital. The patients/participants provided their written informed consent to participate in this study. Written informed consent was obtained from the individual(s) for the publication of any potentially identifiable images or data included in this article.

## Author contributions

TL, Y-TC, R-XL, M-WG, and Q-WL were involved with the conception and design. X-SG and DM revised the manuscript critically. The first draft of the manuscript was written by X-LC and X-ZS. The final draft of the manuscript has been approved by all the authors.

## Funding

This work was supported by grants from the Research Project of China Medical Association of Minorities (2021Z1063-520701) (to Q-WL), the Natural Science Foundation of Tianjin City (20JCQNJC01690) (to X-LC), and the National Natural Science Foundation of China (No. 81801787) (to X-ZS), China Postdoctoral Science Foundation (2018M640238) (to X-ZS).

## Conflict of interest

The authors declare that the research was conducted in the absence of any commercial or financial relationships that could be construed as a potential conflict of interest.

## Publisher's note

All claims expressed in this article are solely those of the authors and do not necessarily represent those of their affiliated organizations, or those of the publisher, the editors and the reviewers. Any product that may be evaluated in this article, or claim that may be made by its manufacturer, is not guaranteed or endorsed by the publisher.
