# Supplementary material for: Case report: Ultrasound-guided multi-site electroacupuncture stimulation for a patient with spinal cord injury
Source: Front Neurol. 2022 Aug 24;13:903207. doi: 10.3389/fneur.2022.903207 (PMC9448914; doi:10.3389/fneur.2022.903207)
Supplement: Supplementary file 1 [file Data_Sheet_1.PDF]

Supplement Table-1 The changes of spinal cord stimulation

	ASIA neurological level	Impairment scale	Motor level	Sensory level
Before treatment	T11	B	T11	T11
After treatment	T11	C	L3	T11

Supplement Table 2 The Slope Vale of Motor and sensory Score

Date	2019/11/11-2020/3/28	2020/3/28-2020/5/15	2020/5/15-2020/11/11
Motor score	0.060946682	0.041666667	0.055003862
sensory score	-0.002863554	0.041666667	0.150171025

Supplement Table 3 The Slope Vale of FIM Score

Date	FIM Score
2019/10/12-2020/4/29	0.013227909
2020/4/29-2020/6/18	0.208198369
2020/6/18-2020/11/1	0.111111111
