# Supplementary material for: Case report: Ultrasound-guided multi-site electroacupuncture stimulation for a patient with spinal cord injury
Source: Front Neurol. 2022 Aug 24;13:903207. doi: 10.3389/fneur.2022.903207 (PMC9448914; doi:10.3389/fneur.2022.903207)
Supplement: Supplementary file 2 [file Data_Sheet_2.PDF]

Supplement Figure 1. The imaging data of patients after rehabilitation

(a)

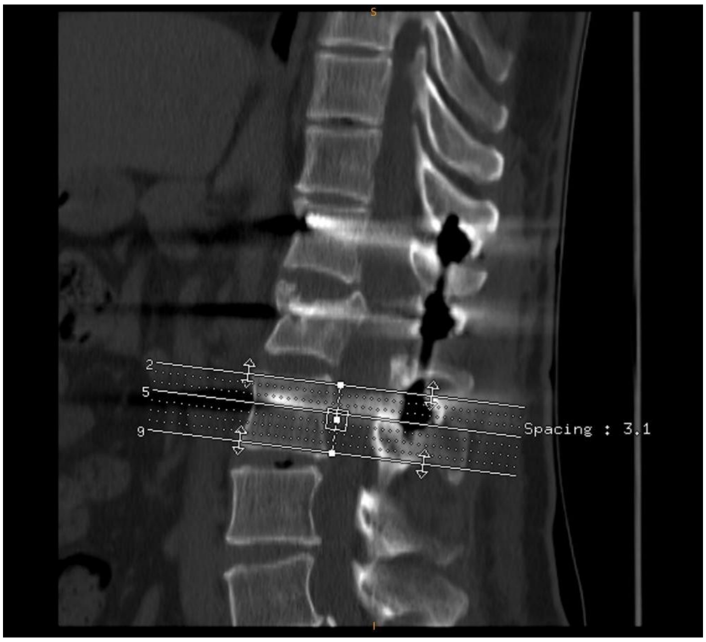

(b)

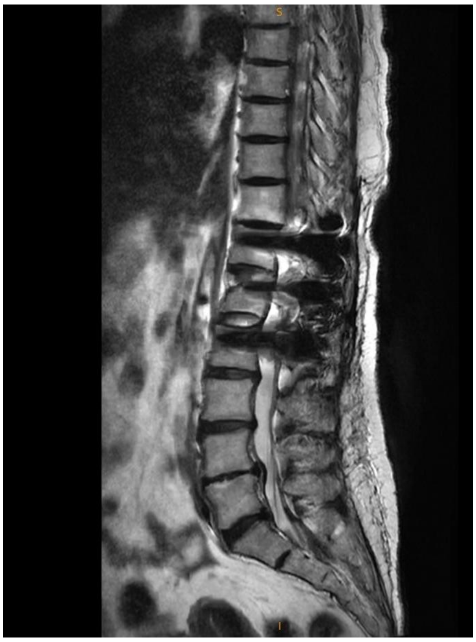

Supplement Figure 1: The imaging data of patients after rehabilitation. (a) Sagittal view of CT scan after treatment. (b) Lumbar in sagittal MR image after treatment.
